# Nutrition and cancer: from prevention to nutritional support, 8th October 2010, Milan

**DOI:** 10.3332/ecancer.2010.205

**Published:** 2010-12-14

Cancer is a disease of genes, which are vulnerable to beneficial and harmful mutations, especially over the long human lifespan. Nutritional factors are important in determining the likelihood of some mutations, as well as in changing gene function even without mutation. Epidemiological and experimental evidence demonstrate that only a small proportion of cancers are inherited; environmental factors are the most important cause of genetic modification. These factors include smoking, infectious agents, radiation, industrial chemicals and pollution and medication. Nutrition, physical activity and body composition can also play a role. Essentially this is good news. It means that a healthy lifestyle can stop cancer before it starts.

In this context, nutrition represents an extraordinary tool to prevent cancer. It is already known that being overweight or obese increases the cancer risk; a healthy diet could reduce this. Furthermore, there is evidence to indicate that bioactives in the diet play an important role in promoting health. Validating health effects of foods and food components represents the new target of nutrition research together with understanding mechanisms through which diet factors could prevent disease.

However, healthy nutritional guidelines are partially known and poorly practiced, others could be established and implemented.

Nutritional improvement requires multiple intervention levels:

- Research
- Health system
- Communication and food industry

The aim of our project ‘The Food Chance’ is devoted to developing nutritional improvement at all levels, taking advantage of our already existing network. The potential benefits of the project include: increased focus on diet and lifespan, motivate positive behaviour change, increased awareness of risk of certain conditions, improve health and healthy ageing, focus on prevention, reduce health care costs, better understanding of the mechanism involved in disease susceptibility.

(a) Research: different fields of research are involved in the project. Basic research is dedicated to identifying mechanisms and markers of obesity cancer promotion, the fundamental activities will include:

1. The role of caloric intake and fat development in tumorigenesis.
2. The effect of obesity on epithelial and haematopoietic mouse stem cell compartment.
3. The investigation into the epigenome of caloric restriction and obesity in mouse stem cells.
4. The identification of specific epigenetic modifications induced by fat in mouse stem cells and validation in humans.

Furthermore, a metabolism laboratory will study the following:

- genetics of the metabolism of bioenergetics
- aerobic metabolism and oxidative stress
- mitochondrial cellular crosstalk
- mitochondrial role in aging and cancer.

Finally, the dietary components that could have beneficial effects on health, i.e. in preventing chronic diseases or ameliorating general wellness will be investigated by carrying out a literature analysis and diet intervention trials in which subjects are assigned to a dietary treatment intervention and their outcomes measured.

The direct effect of diet on a range of disease states can be assessed. Research focuses mainly on dietary macro and micronutrients and their effects on chronic disease (cardiovascular disease, cancer, diabetes type 2).

(b) Health system: represents a fundamental area for any prevention policy. In the project, we plan many activities in our hospital strongly linked with the other areas: dietary intervention studies program on healthy volunteer and patients release of informative material for hospital staff, patients and visitors canteen and hospital kitchen reorganization following best guidelines, specific hospital staff training.

(c) Communication and food industry: by providing clear recommendations of the efficacy of particular food-related components to confer protection against target diseases and reliable estimates of active compound levels in a directory of foods; this programme will result in a recipe for promoting health through diet that will appeal to the diversity of tastes of consumers.

## Figures and Tables

**Table 1:**
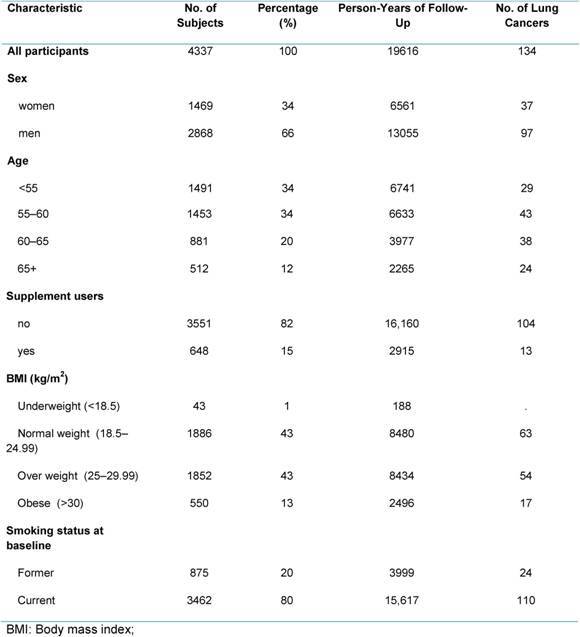
Demographic characteristic, BMI and smoking habits in COSMOS participants

**Table 2:**
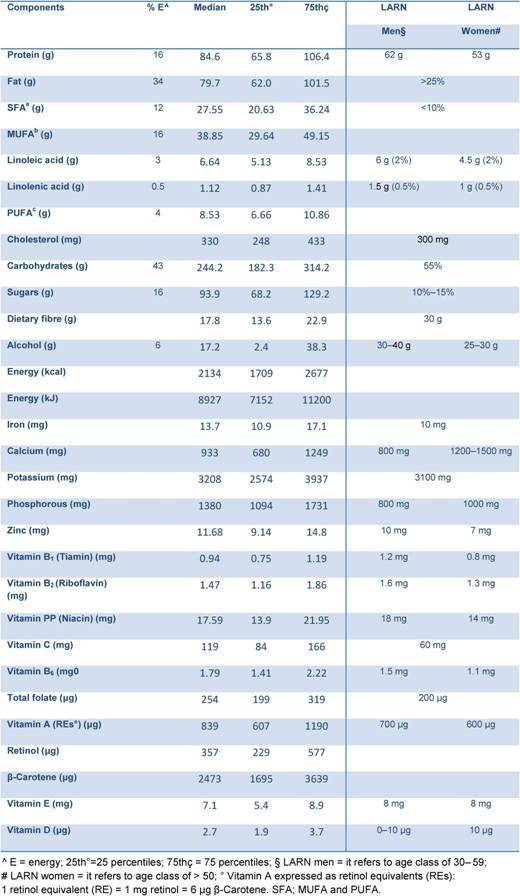
Median energy and nutrients intake from food in all study population (4337) compared to the national recommendations [4]

**Table 3:**
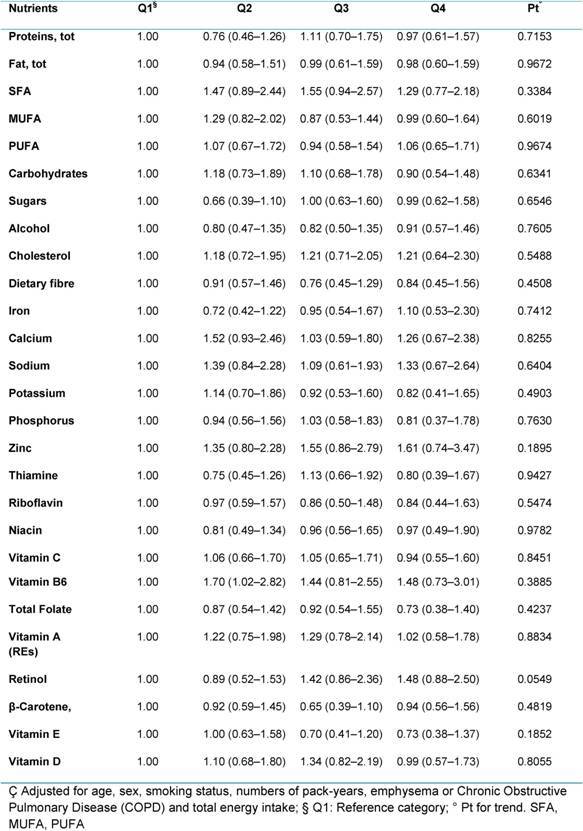
Hazard ratios (HRs) (95% CI)^ç^ for lung cancer, according to quartiles of consumption of specific nutrients for all participants (4337)

## References

[1] Zapas JL, Karakozis S, Kirkpatrick JR (1998). Prophylactic jejunostomy: a reappraisal. Surgery.

[2] Wakefield SE, Mausell NJ, Baigrie RJ, Dowling BL (1995). Use of a feeding jejunostomy after esophagogastric surgery. Br J Surg.

[3] Pescovitz MD, Mehta PL, Leapman SB, Milgrom ML, Jindal RM, Filo RS (1995). Tube jejunostomy in liver transplant recipients. Surgery.

[4] Swails WS, babineau TJ, Ellis FH, Kenler AS, Forse RA (1995). The role of enteral jejunostomy feeding after esophagogastrectomy: a prospective, randomized study. Dis Esophagus.

[5] Sarr MG (1988). Needle catheter jejunostomy: an unappreciated and misunderstood advance in the care of Patients after major abdominal operations. Mayo Clin Proc.

[6] Dent D, Kusalk KA, Minarol G (1993). Risk of abdominal septic complications after feeding jejunostomy placemen in Patients undergoing splenectomy for trauma. Am J Surg.

[7] Dahn MS (1994). Shifting ground: enteral versus parenteral nutrition in critically ill Patients. Nutr Clin Pr.

[8] Eddy VA, Snell JE, Morris JA (1996). Analysis of complications and long-term outcome of trauma Patients with needle catheter jejunostomy. Am Surg.

[9] Sarr MG (1999). Appropriate use, complications and advantages demonstrated in 500 consecutive needle catheter jejunostomies. Br J Surg.

