# Supplementary material for: Nutrition and cancer: from prevention to nutritional support, 8th October 2010, Milan
Source: Ecancermedicalscience. 2010 Dec 14;4:205. doi: 10.3332/ecancer.2010.205 (PMC3234016; doi:10.3332/ecancer.2010.205)
Supplement: Related texts

# Sub article: Nutrition and surgery: state of the art

**Published:** 2010

**Authors:** R Biffi

**Affiliations:** Division of Abdomino-Pelvic Surgery, European Institute of Oncology, Milano, Italy

## Background

Over the years, enteral nutrition (EN) has gained considerable popularity due to its favourable effects on the digestive tract, its lower cost and rate of complications compared to parenteral nutrition.

However, clinicians are often faced with contradictory data and difficult decision making when having to determine the optimal modalities of EN administration. An example of such a difficulty is the precise definition of the expected morbidity rate of needle catheter jejunostomy (NCJ). Some findings suggest that NCJ is associated with a significant rate of early and late complications; in some instances, they may be life threatening and require surgical intervention. In this scenario, the role of NCJ in providing nutritional therapy to depleted patients undergoing major gastrointestinal procedures should be re-examined.

We prospectively evaluated the rate and type of early and late complications associated with NCJ in patients undergoing surgical treatment for upper gastrointestinal malignancies.

## Methods

Three hundred and eighty consecutive patients underwent NCJ implant according to Delany’s method at the end of their scheduled surgical procedure (total/subtotal gastrectomy = 242; duodenopancreatectomy = 45; esophagectomy = 78; other procedures = 15). The EN program was started on post-operative day (pod) 1 in the surgical ICU with 15 ml/h and progressed to the optimal goal of 80 ml/h by post-operative day 5, using a nutritionally complete liquid polymeric diet providing 1 kcal/ml.

Clinical examination and adverse gastrointestinal symptoms were recorded on a daily basis.

Short-term complications directly attributable to the NCJ were defined as tube leakage with intra-abdominal or intraparietal spillage, intra-abdominal abscess, small bowel obstruction at the catheter site, tube blockage or dislodgement or soft tissue infection. The NCJ was always removed in the outpatient clinic after hospital discharge. Regular follow-up visits were conducted in all cases until the patient died or the study was closed, in order to detect long-term complications, including operations to correct adhesions or small bowel obstruction.

## Results

All the Patients received the EN program for a mean of 14 pod (range: 8–46); a single short-term complication of the NCJ has been observed in this series (tube blockage; third pod). An easy resolution has been obtained with percutaneous replacement under x-ray control. No late complications have been detected after a mean follow-up of 24.01 months (range: 2–42).

## Discussion

Extensive effort has been made to improve outcome of patients undergoing major surgical procedures of the upper gastrointestinal tract, and nutrition support is now recognized an essential component of the clinical management of these patients. The ability to deliver nutrition enterally after an operation has been a significant advance and the NCJ gained popularity, leading some investigators to recommend prophylactic jejunostomy at the time of any high-risk abdominal operation, emphasizing the substantial safety and the relatively low cost of this additional procedure. However, a few studies investigated prospectively the rate of complications arising from the NCJ, and limited data are provided from the medical literature coming from randomized trials. Zapas *et al* [1] carried out a benefit/risk analysis, comparing complication rate, avoidance of parenteral nutrition and discontinuation of jejunostomy feeding in 92 patients [1], concluding that the benefit/risk ratio is low, mainly due to the significant rate of complications related to the placement procedure. Although many reports of the use of NCJ have shown very low major complication rate, ranging from 0 to 2% [2,3,5–7] in different clinical setting, Eddy *et al* [8] published a paper analysing retrospectively their experience in 122 patients observed in a Level I Trauma Centre, indicating 14% of short-term complications (8% serious) independent from injury severity score, trauma mechanism and site of injury and surgeon. The results of this series lead them to postulate that NCJ may be, at last in trauma patients, associated with a significant rate of potentially disastrous complications. The weakness of this study are those common to any retrospective review; moreover, it is possible that trauma patients are more prone than general surgery patients to develop serious complications, particularly if an etiologic role for dehiscence of jejunostomy and the formation of an enterocutaneous fistula at the exit site of the catheter could be attributed to the shrinkage of the oedema creating a space between the catheter, the bowel wall and the abdominal wall thus facilitating the leak of the jejunostomy.

A small prospective randomized study has investigated the safety and efficacy of enteral feeding by jejunostomy in patients undergoing elective esophagogastrectomy [4], comparing the clinical outcomes of 25 patients, 13 receiving immediate post-operative enteral feeding and 12 given a routine diet advancement and no feeding jejunostomy. Again, there were no major complications related to placement of the feeding tube, and there was a trend for patients fed via jejunostomy to have a shorter hospital stay, thus confirming that early enteral feeding by jejunostomy tube, placed at the time of esophagogastrectomy, is a safe means of providing post-operative nutrition support in selected patients.

As the benefits of early enteral feeding over total parenteral nutrition in terms of reduction of septic morbidity and lower costs are indisputable and demonstrated by a number of randomized trials, a possible solution to overcome the NCJ-related morbidity could be the reconsideration of the NCJ as the sole way to provide effective enteral nutrition in the post-operative period, in favour of an intrajejunal feeding initiated via a nasally passed tube. No data coming from well-designed randomized trials are presently available about this specific issue; nevertheless, the compliance of the patients towards this approach should be considered as a possible, major limiting factor, as at least 27% of nasally passed tubes for enteral nutrition are expected to be voluntarily or accidentally removed within a few days after placement [8].

Finally, a large series (500 consecutive cases) recently published by a monocentric experience [9] showed only three major complications: small bowel obstruction and pneumatosis intestinalis in two patients, respectively; minor complications, including diarrhoea and abdominal distension, occurred in 15% of patients, whereas subcutaneous infection at the insertion site and catheter occlusion precluding use have been detected in just 1% of cases. The results of our prospective non-randomized study agree with these data: complications are essentially confined to diarrhoea and abdominal distension and can be controlled in the vast majority of the cases with a strict adherence to standardized treatment protocols and appropriate reduction of the nutrient flow. In addition, long-term morbidity is very rare and should not be considered as a significant factor in the decision to perform this procedure.

## References

[1] Zapas JL, Karakozis S, Kirkpatrick JR (1998). Prophylactic jejunostomy: a reappraisal. Surgery.

[2] Wakefield SE, Mausell NJ, Baigrie RJ, Dowling BL (1995). Use of a feeding jejunostomy after esophagogastric surgery. Br J Surg.

[3] Pescovitz MD, Mehta PL, Leapman SB, Milgrom ML, Jindal RM, Filo RS (1995). Tube jejunostomy in liver transplant recipients. Surgery.

[4] Swails WS, babineau TJ, Ellis FH, Kenler AS, Forse RA (1995). The role of enteral jejunostomy feeding after esophagogastrectomy: a prospective, randomized study. Dis Esophagus.

[5] Sarr MG (1988). Needle catheter jejunostomy: an unappreciated and misunderstood advance in the care of Patients after major abdominal operations. Mayo Clin Proc.

[6] Dent D, Kusalk KA, Minarol G (1993). Risk of abdominal septic complications after feeding jejunostomy placemen in Patients undergoing splenectomy for trauma. Am J Surg.

[7] Dahn MS (1994). Shifting ground: enteral versus parenteral nutrition in critically ill Patients. Nutr Clin Pr.

[8] Eddy VA, Snell JE, Morris JA (1996). Analysis of complications and long-term outcome of trauma Patients with needle catheter jejunostomy. Am Surg.

[9] Sarr MG (1999). Appropriate use, complications and advantages demonstrated in 500 consecutive needle catheter jejunostomies. Br J Surg.

# Sub article: DIANA 5 trial at the IEO: the role of the dietician

**Published:** 2010

**Authors:** E Zagallo

**Affiliations:** Dietetic and Clinical Nutrition Unit, European Institute of Oncology, Milan, Italy

The Diana 5 (Diet and Androgens) trial is a multicentric study promoted by the Istituto Nazionale dei Tumori of Milan that involves several centres in Italy, including the European Institute of Oncology.

It is an open-label randomized trial based on a change in diet and physical activity in order to reduce the risk of breast cancer recurrence after surgery in individuals receiving conventional cancer management.

Previous Diana trials showed that a diet that keeps low serum insulin levels promotes weight loss, testosterone and insulin decrease and reduces oestrogens and IGF-I bioavailability both in healthy women and in breast cancer survivors [1–2]. Other studies showed that an increase in physical activity is associated with a significant reduction in insulin resistance, body weight and risk of some cancer types [3–7]. The WINS (Women’s Intervention Nutrition Study) study suggests an improvement in relapse-free survival of breast cancer patients through a healthy diet [8].

Eligibility criteria are: women, ages 35–70 years, with invasive breast carcinoma, diagnosed in the last five years, with no recurrences or other tumours.

Women with hormone negative breast cancer can be included in the study independently of metabolic characteristics.

Women with breast cancer sensitive to hormones can be included in the study if at least one of the following endocrine--metabolic characteristics is present:

- Levels of serum testosterone: ≥ 0.4 ng/ml.
- Levels of serum insulin: ≥7 μU/ml (50 pmol/l).
- Metabolic syndrome (three of the following factors):
  1. Glycaemia ≥ 100 mg/dl (or therapy for hyperglycaemia).
  2. Triglycerides ≥150 mg/dl (or therapy for high triglycerides).
  3. HDL <50 mg/dl (or therapy for hypercholesterol).
  4. Waist circumference ≥ 88 cm.
  5. Blood pressure ≥ 130/85 mmHg (or therapy for hypertension).

This trial lasts one year and will have a follow-up of five years.

Women are randomized to the intervention or control groups.

Women of both groups receive the ‘cancer prevention decalogue’ of the World Cancer Research Fund [9].

Patients in the intervention group get further information within cooking classes, gymnastics classes, common meals and meetings.

The Diana 5 trial started at IEO in 2009 and patient recruitment is currently in progress. The trial requires a multidisciplinary approach, involving an oncologist, a biologist, a dietician and a research nurse.

The dietician has to perform an anthropometric evaluation, which includes also the bioimpedenziometric analysis (with the use of Tanita^®^ scale), at the beginning of the study and during follow-up.

The dietician also has to illustrate and discuss with the patients the main points of the World Cancer Research Fund recommendations.

The dietician contact each patient by telephone 10 times during the first year of the study to get information about physical activity and food eaten during the previous day.

The 24-h dietary recall does not include questions about food servings, but only questions about type of food consumed. Food intake-recall strategy requires the presence of adequately trained staff.

The dietician is important in the multidisciplinary team to make an accurate anthropometric evaluation, an educational activity at the beginning and during the first year of the study and dietary intake recording through 24-h dietary recall [10].

## References

[1] Berrino F, Bellati C, Secreto G, Camerini E, Pala V, Panico S, Allegro G, Kaaks R (2001). Reducing bioavailable sex hormones through a comprehensive change in diet: the diet and androgens (DIANA) randomized trial. Cancer Epidemiol Biomarkers Prev.

[2] Kaaks R, Bellati C, Venturelli E, Rinaldi S, Secreto G, Biessy C, Pala V, Sieri S, Berrino F (2003). Effects of dietary intervention on IGF-I and IGF-binding proteins, and related alterations in sex steroid metabolism: the Diet and Androgens (DIANA) Randomised Trial. Eur J Clin Nutr.

[3] Holmes MD, Chen WY, Feskanich D, Kroenke CH, Colditz GA (2005). Physical activity and survival after breast cancer diagnosis. JAMA.

[4] Holick CN, Newcomb PA, Trentham-Dietz A, Titus-Ernstoff L, Bersch AJ, Stampfer MJ, Baron JA, Egan KM, Willett WC (2008). Physical activity and survival after diagnosis of invasive breast cancer. Cancer Epidemiol Biomarkers Prev.

[5] Irwin ML, Yasui Y, Ulrich CM, Bowen D, Rudolph RE, Schwartz RS, Yukawa M, Aiello E, Potter JD, McTiernan A (2003). Effect of exercise on total and intra-abdominal body fat in postmenopausal women: a randomized controlled trial. JAMA.

[6] McTiernan A, Tworoger SS, Rajan KB, Yasui Y, Sorenson B, Ulrich CM, Chubak J, Stanczyk FZ, Bowen D, Irwin ML, Rudolph RE, Potter JD, Schwartz RS (2004). Effect of exercise on serum androgens in postmenopausal women: a 12-month randomized clinical trial. Cancer Epidemiol Biomarkers Prev.

[7] McTiernan A, Tworoger SS, Ulrich CM, Yasui Y, Irwin ML, Rajan KB, Sorensen B, Rudolph RE, Bowen D, Stanczyk FZ, Potter JD, Schwartz RS (2004). Effect of exercise on serum estrogens in postmenopausal women: a 12-month randomized clinical trial. Cancer Res.

[8] Chlebowski RT, Blackburn GL, Thomson CA, Nixon DW, Shapiro A, Hoy MK, Goodman MT, Giuliano AE, Karanja N, McAndrew P, Hudis C, Butler J, Merkel D, Kristal A, Caan B, Michaelson R, Vinciguerra V, Del Prete S, Winkler M, Hall R, Simon M, Winters BL, Elashoff RM (2006). Dietary fat reduction and breast cancer outcome: interim efficacy results from the Women’s Intervention Nutrition Study. J Natl Cancer Inst.

[9] World cancer Research Fund. WCRF/ AICR (2007). Food, Nutrition and the Prevention of Cancer: a Global Perspective. www.Dietandcancerreport.org.

[10] ANDID. D.M. n. 744, 1994 Sep. Dietitian Professional Profile.

# Sub article: Is there a role for vitamin D in cancer?

**Published:** 2010

**Authors:** S Gandini

**Affiliations:** Division of Epidemiology, European Institute of Oncology, Milan, Italy

Calcitriol (1,25(OH)D), the biologically active form of vitamin D, has been shown to be an anti-proliferative, pro-differentiation, pro-apoptotic agent and an inhibitor of cell migration. The vitamin D receptor (VDR) is a crucial mediator for the cellular effects of vitamin D and additionally interacts with other cell-signalling pathways that influence cancer development. Alterations in vitamin D receptor expression and in the synthesis and catabolism of vitamin D metabolites are involved in the growth regulation of tumours, thus, compromising calcitriol sensitivity and signalling.

Cultured melanoma cells can synthesise 1,25(OH)D from 25-hydroxyvitamin D (25(OH)D), express the VDR, and the anti-proliferative and pro-differentiation effect of 1,25(OH)D have been shown in human malignant melanoma (MM) cells. The 1,25(OH)D molecule has been shown to induce apoptosis in a human MM cell line *in vitro*, and an inhibitory effect on the spread of MM cells has recently been demonstrated *in vitro*.

## Preliminary results

Epidemiological data indicate that vitamin D deficiency is relatively common in Europe. In an Italian study, we found that 85% of the participants had insufficient level of circulating 25(OH)D. We show that vitamin D supplementation was associated with a significant reduction (7%) in total mortality in healthy subjects. When we looked at cancer risk, we showed consistent inverse relationship between serum 25-hydroxyvitamin D levels and colorectal cancer incidence. For breast cancer, results were heterogeneous by study design; in fact, serum 25(OH)D level are measured after the diagnosis of cancer and a reverse causation problem can occur.

We also showed, through a meta-analysis of the association between the two most studied VDR polymorphisms (*Fok*I and *Bsm*I), that these polymorphisms might modulate the risk of cancer of breast, skin and prostate and possibly affect cancer risk at any site in Caucasians.

A cohort study investigated the hypothesis that higher vitamin D levels reduce the risk of relapse from melanoma. The survival analysis showed that higher 25(OH)D levels, at diagnosis, were associated with lower Breslow thickness and were independently protective of relapse and death. There was also evidence of interaction between *Bsm*I and serum 25OHD levels on relapse.

## Aims of MelaViD trial and ancillary studies

In early supplementation trials, the lack of effect on cancer incidence has been attributed to insufficient vitamin D supplementation, stressing the need to better study vitamin D bioavailability.

In order to assess whether vitamin D supplementation could improve prognosis of Cutaneous Malignant Melanoma (CMM), we planned an Italian multi-centre trial in stage II resected melanoma patients, evaluating the effect of vitamin D supplementation and monitoring changes in 25(OH)D.

We intend also to study in detail the biology of VDR on vitamin D metabolism and in relation to CMM prognosis, looking at the association between 25(OH)D and VDR polymorphisms with Breslow thickness, the most important prognostic factor of CMM. In fact, tumour thickness could be the result of an effect on cell proliferation, and this study could give more information on functional effects of VDR and on the mechanisms that potentially make the vitamin D important in cancer development. We will study gene–gene interactions and changes in vitamin D metabolites, investigating how metabolism and biology of vitamin D interacts with environmental, anthropometrical and nutritional factors and contributes to cancer progression.

Some of the findings from this study will be of large interest also for a wide spectrum of cancers.

## References

[1] Berrino F, Bellati C, Secreto G, Camerini E, Pala V, Panico S, Allegro G, Kaaks R (2001). Reducing bioavailable sex hormones through a comprehensive change in diet: the diet and androgens (DIANA) randomized trial. Cancer Epidemiol Biomarkers Prev.

[2] Kaaks R, Bellati C, Venturelli E, Rinaldi S, Secreto G, Biessy C, Pala V, Sieri S, Berrino F (2003). Effects of dietary intervention on IGF-I and IGF-binding proteins, and related alterations in sex steroid metabolism: the Diet and Androgens (DIANA) Randomised Trial. Eur J Clin Nutr.

[3] Holmes MD, Chen WY, Feskanich D, Kroenke CH, Colditz GA (2005). Physical activity and survival after breast cancer diagnosis. JAMA.

[4] Holick CN, Newcomb PA, Trentham-Dietz A, Titus-Ernstoff L, Bersch AJ, Stampfer MJ, Baron JA, Egan KM, Willett WC (2008). Physical activity and survival after diagnosis of invasive breast cancer. Cancer Epidemiol Biomarkers Prev.

[5] Irwin ML, Yasui Y, Ulrich CM, Bowen D, Rudolph RE, Schwartz RS, Yukawa M, Aiello E, Potter JD, McTiernan A (2003). Effect of exercise on total and intra-abdominal body fat in postmenopausal women: a randomized controlled trial. JAMA.

[6] McTiernan A, Tworoger SS, Rajan KB, Yasui Y, Sorenson B, Ulrich CM, Chubak J, Stanczyk FZ, Bowen D, Irwin ML, Rudolph RE, Potter JD, Schwartz RS (2004). Effect of exercise on serum androgens in postmenopausal women: a 12-month randomized clinical trial. Cancer Epidemiol Biomarkers Prev.

[7] McTiernan A, Tworoger SS, Ulrich CM, Yasui Y, Irwin ML, Rajan KB, Sorensen B, Rudolph RE, Bowen D, Stanczyk FZ, Potter JD, Schwartz RS (2004). Effect of exercise on serum estrogens in postmenopausal women: a 12-month randomized clinical trial. Cancer Res.

[8] Chlebowski RT, Blackburn GL, Thomson CA, Nixon DW, Shapiro A, Hoy MK, Goodman MT, Giuliano AE, Karanja N, McAndrew P, Hudis C, Butler J, Merkel D, Kristal A, Caan B, Michaelson R, Vinciguerra V, Del Prete S, Winkler M, Hall R, Simon M, Winters BL, Elashoff RM (2006). Dietary fat reduction and breast cancer outcome: interim efficacy results from the Women’s Intervention Nutrition Study. J Natl Cancer Inst.

# Sub article: Nutritional support in cancer

**Published:** 2010

**Authors:** D Alterio

**Affiliations:** Division of Radiotherapy, European Institute of Oncology, Milan, Italy

In a contest of multidisciplinary approach, an adequate nutritional support should be considered of fundamental importance as supportive therapy in patients treated with radiotherapy for malignant tumours. Radiation therapy, in fact, produces an acute mucosae inflammation and, after the end of treatment, a chronic soft tissues fibrosis, which could produce a transient or a definitive alteration of food intake and/or food absorption. For these reasons, patients taking radiation therapy should be evaluated prior to treatment and offered specialized nutritional counselling in order to optimize the treatment and prevent radiation-related side effects. In head and neck and thoracic regions, acute radiotherapy-related dysphagia is due to the inflammatory process of irradiated tissue (oral cavity, pharyngeal and oesophagus mucosa) with the development of mucositis, oedema and pain. After the end of treatment, late dysphagia might be due to soft tissues fibrosis, chronic lymphedema and damage to neural tissue. Any hindrance to oral food intake should be therefore prevented and adequately supported. During the first phases of pharyngeal mucositis, oral supplementation is used when oral intake is still permitted. Subsequently, when patients cannot adequately swallow, feeding by tube is usually given. Tube feeding nutrition can be performed by a nasogastric tube (generally not preferred and used only for short period because of its negative cosmetic impact and because of the high risk of mucosal injury, sinusitis and aspiration) or by gastrostomy (percutaneous endoscopic gastrostomy – PEG).

Apart from the patient’s quality of life, the clinically relevant negative impact of dysphagia and the subsequent weight loss in response to anti-cancer therapies is well known [4, 10]. These results could be explained by the fact that an inadequate patient nutritional status may require temporary or definitive treatment interruption. For this reason, in recent years, effort has been made to prevent and to ameliorate the nutritional deficiency in these patients. Despite of these efforts, a range varying from 10% to 70% of patients suffered weight loss of more than 10% when undergoing concomitant chemoradiation to head and neck region [11, 1–3, 8, 9). Moreover, a recent review, focusing on nutritional support in head and neck cancer patients [5], showed that dietary counselling performed by a professional dietician resulted in less weight loss and improved oral intake compared to when dietary counselling was given by a nurse and/or from general advice or a nutritional booklet or not at all. These results were also confirmed in a recent randomized trial in which patients treated with intensive nutrition intervention following the American Dietetic Association medical nutrition therapy protocol resulted to have a higher intake of calories and proteins compared to patients advised generally or with booklets [7].

Radiation therapy to the abdominal or pelvic region has different consequences. These patients may experience radiation-induced enteritis resulting in intestinal malabsorption. Also, for gastrointestinal tumours, a recent study, although performed with a limited number of patients, showed that the worsening of nutritional status resulted to be associated with worse results of anti-cancer therapies [6].

In conclusion, controlled clinical trials and Institutional guidelines are strongly recommended in order to optimize the nutritional assessment and intervention for oncologic patients undergoing radiation therapy.

## References

[1] Adelstein DJ, Saxton JP, Lavertu P (1997). A phase III randomized trial comparing concurrent chemotherapy and radiotherapy with radiotherapy alone in resectable stage III and IV squamous cell H&N cancer: preliminary results. Head Neck.

[2] Calais G, Alfonsi M, Bardet E (1999). Randomized trial of radiation therapy versus concomitant chemotherapy and radiation therapy of advanced-stage oropharynx carcinoma. J Natl Cancer Inst.

[3] Calais G, Bardet Sire C (2004). Radiotherapy with concomitant weekly docetaxel for stage III/IV oropharynx carcinoma. Results of the 98-02 GORTEC phase II trial. Int J Radiat Oncol Biol Phys.

[4] Dewys WD, Begg C, Lavin PT (1980). Prognostic effects of weight loss prior to chemotherapy in cancer patients. Am J Med.

[5] Garg S, Yoo J, Winquist E (2009). Nutritional support for head and neck cancer patients receiving radiotherapy: a systematic review. Supp Care Cancer.

[6] Hill A, Kiss N, Hodgson B, Crowe TC, Walsh AD (2010). Association between nutritional status, weight loss, radiotherapy treatment, toxicity and outcome in gastrointestinal cancer patients. Clinical Nutrition.

[7] Isenring EA, Bauer JD, Capra S (2007). Nutritional support using the American Dietetic Association medical nutrition protocol for radiation oncology patients improves dietary intake compared with standard practice. J Am Diet Assoc.

[8] Langenberg M, Terhaard CH, Hordijk GJ (2004). Simultaneous radio- and chemotherapy for squamous cell carcinoma of the head and neck in daily clinical practice: 5 years experience in a University Hospital. Clin Otolaryngol Allied Sci.

[9] Lin A, Jabbari S, Worden FP (2005). Metabolic abnormalities associated with weight loss during chemoirradiation of head and neck cancer. Int J Radiat Oncol Biol Phys.

[10] Vigano A, Watanabe S, Bruera E (1994). Anorexia and cachexia in advanced cancer patients. Cancer Surv.

[11] Wiggenraad RGJ, Flierman L, Goossens A (2007). Prophylactic gastrostomy placement and early tube feeding may limit loss of weight during chemoradiotherapy for advanced head and neck cancer, a preliminary study. Clin Otolaryngol.

# Sub article: Anti-oxidants and other bioactive compounds in the Mediterranean diet and cancer prevention

**Published:** 2010

**Authors:** C Galli

**Affiliations:** Department of Pharmacological Science, University of Milan, Italy

The uncontrolled production of free radicals, activated by oxidative stress, is considered a relevant factor in carcinogenesis [6]. As a consequence, interest in the possible roles of antioxidants (AO) as components of the diet or as supplements, as well as of natural bioactive compounds endowed of modulatory activity with respect to these events [9] has greatly grown in the last years. In this respect, the Mediterranean diet, especially the one that has been defined in greater details, i.e. that typical of the Southern European countries of Europe, is characterized by a high consumption of food items, i.e. vegetables and fruits, that are rich in AO. This has provided the basis of studies of the relationships (correlations/associations) between dietary AO and incidence of cancer. It should be noted, however, that the typical Mediterranean diet is characterized also by other features (e.g. low contents of saturated fatty acids, animal proteins, simple sugars and appreciable consumption of fish) that may contribute to affect the possible relationships, thus making more complex the interpretation of the results.

Experiments *in vitro* and in animal models may provide some information on the mechanisms that involved in the modulation of carcinogenesis by selected compounds, but the relevance of these approaches to investigate the situation in humans in essence is rather limited.

A number of studies have been carried out in humans, concerning the correlations/associations between estimates of AO consumption, or AO administrations or endogenous levels of AO, on one side, and cancer incidence or effects of treatments, on the other. Various study designs have been applied, from observational studies, e.g. case-control, cohort, to controlled randomized clinical trials, on rather different population sizes and of different duration, but the results are somewhat contradictory.

It should be considered, however, that relevant unresolved issues (both conceptual and practical) make the approach to this research area rather complex and difficult:

- Difficulties in the evaluation of the oxidative stress status ‘*in vivo*’, due to the lack of fully validated ‘biomarkers’ [2] generally applicable to chronic diseases, e.g. cardiovascular disease.
- The extremely high number of compounds endowed of AO and biological activities in many foods, up to 25,000 different compounds [1] and their variability (due to genetic and environmental factors) make their identification and quantification in complex diets rather difficult.
- In addition, research aimed to investigate the modulation of processes, such as carcinogenesis, following the intakes of these compounds, face a number of problems dependent upon the study design.
  1. Intake of foods rich in various AO: bioavailability, metabolism, interactions between the various compounds, from the intestinal absorption (interactions with transporters and transcription factors) to the functional ones (potentiation or inhibition) and doses required to reach active concentrations in the body.
  2. Studies with pure compounds based on a ‘pharmacological’ approach (i.e. high doses of a single compound), on one side, may allow the evaluation of the role of the compound at study, but on the other, these studies are based on strategies that are not biologically (and nutritionally) correct: pro-oxidant effects of high doses, interference with the bioavailability and the activities of other AO in the body or with endogenous AO systems.

Key aspects in the design of these studies, in addition to the identification and quantification of the AO compounds in foods, are the assessment of the AO potential of the subject (e.g. plasma anti-oxidant capacity), of the plasma levels of the compounds or at least of the main AO (vitamins or non-vitamins, e.g. flavonoids, phenols, carotenoids, etc.) and of their major metabolites. These data need to be correlated, on one side, with the estimated (epidemiological studies) or the administered (controlled studies) amounts of AO, and, on the other, with pathophysiological data of clinical relevance. In addition, it should be considered that AO, even when poorly absorbed, may act at the gastrointestinal site, where they reach concentrations considerably higher than in the rest of the body and where they may play important roles in protecting the gastrointestinal tract itself from oxidative damage and in delaying the development of stomach, colon and rectal cancers [3].

On the basis of these considerations and of the critical evaluation of the vast literature that concerns major human studies and meta-analyses available, with attention to the situation in the Mediterranean area, and considering also the strength of the evidence, the following conclusions can be drawn:

- Convincing evidence: Several studies, especially those carried out with a case-control protocol [8], provide convincing evidence that dietary AO, especially those in the Mediterranean diet [5], exert protective effects on certain cancers, e.g. prostate, oesophagus, colon, bladder, especially in the case of lycopene and tocopherols.
- Probable evidence: A certain number of studies provide probable evidence of protective effects, especially of betacarotene and vitamin C on breast cancer risk in post-menopausal women [7].
- No effect or insufficient evidence: The majority of the studies, meta-analyses and reviews, indicate that there is no effect or insufficient evidence of a relationship between dietary AO and cancer prevention.

In general, the main AO involved in the protective effects are vitamins E, D and C, and carotenoids, especially lycopene. On the other side, since the total anti-oxidant status declines during cancer treatment [4], supplementation of AO in association with the pharmacological treatments appears to be justified. There is also a general agreement that more adequate intervention and observational studies (number of subjects, duration of the studies) are needed to reach conclusions. It should be added also that studies with single and pure compounds do not generally lead to favourable effects.

It is generally agreed that the available knowledge indicates that the efficacy of natural AO is greater when consumed as component foods in the diet. There is also the consensus that recommendations to increase fruit and vegetable consumption are justified, since they contribute to the prevention of a number of chronic diseases including cancer. In addition, this dietary strategy appears to be associated with complementary healthy modifications of the diet (reduction of calories, of the consumption of meat and of fat intakes while favouring a healthier fat composition) and facilitates lifestyle improvements, e.g. weight control also through physical activity.

## References

[1] Carlsen MH, Halvorsen BL, Holtel K (2010). The total antioxidant content of more than 3100 foods, beverages, spices, herbs and supplements used worldwide. Nutrition Journal.

[2] Halliwell B (2000). Lipid peroxidation, antioxidants and cardiovascular disease: how should we move forward?. Cardiovascular Research.

[3] Halliwell B, Zhao K, Whiteman M (2000). The gastrointestinal tract: a major site of antioxidant action?. Free Rad Res.

[4] Ladas EJ, Jacobson JS, Kennedy DD (2004). Antioxidants and cancer therapy: a systematic review. J Clin Oncol.

[5] La Vecchia C, Altieri A, Tavani A (2001). Vegetables, fruit, antioxidants and cancer: a review of Italian studies. Eur J Nutr.

[6] Lopez-Lazaro NM (2007). Excessive superoxide anion generation plays a key role in carcinogenesis. Int J Cancer.

[7] Nagel G, Linseisen J, van Gills CH (2010). Dietary b-carotene, vitamin C and E intake and breast cancer risk in the European Perspective Investigation into Cancer and Nutrition (EPIC). Breat Cancer Res Treat.

[8] Riboli E, Norat T (2003). Epidemiologic evidence of the protective effect of fruit and vegetables on cancer risk. Am J Clin Nutr.

[9] Williams RJ, Spencer JP, Rice-Evans C (2004). Flavonoids : Antioxidants or signalling molecules?. Free Radical Biology & Medicine.

# Sub article: Components of Mediterranean diet in the prevention of cancer

**Published:** 2010

**Authors:** C La Vecchia

**Affiliations:** Istituto di Ricerche Farmacologiche ‘Mario Negri’ Milano e Università degli Studi di Milano, Milano, Italy

‘Mediterranean diet’ has been associated with reduced risk not only of cardiovascular disease but also of cancer at various sites. Selected aspects of the Mediterranean diet have been related to cancer risk; a series of case-control studies conducted in Italy since the early 1980s on over 15,000 cases of 20 cancer sites, and a similar number controls. For most epithelial cancers, and particularly for digestive tract cancers, the risk decreased with increasing vegetable and fruit consumption, with relative risks between 0.3 and 0.7 for the highest versus the lowest tertile. A number of vitamins and other micronutrients or food components (including carotenoids, lycopene and flavonoids) showed an inverse relation with cancer risk. With reference to flavonoids, our data provided support for a protective role of flavanones on upper aerodigestive tract, proanthocyanidins on gastric cancer, flavonols and proanthocyanidins on colorectal, flavonols and flavones on breast, and isoflavones on ovarian cancers. However, the components responsible for the favourable effect of a diet rich in vegetables and fruit remain largely undefined. Fish, and consequently, a diet rich in n-3 fatty acids also tended to be favourable diet indicators. In contrast, subjects reporting frequent red meat intake showed elevated risks for several common neoplasms. Whole grain food (and hence fibre) intake was related to reduced risk of several cancers, particularly of the upper digestive tract, but refined grain intake and, consequently, glycaemic load and glycaemic index were associated with increased risk of different types of cancers. Olive oil—the major common characteristic of diet in various parts of the Mediterranean—is a major source of monounsaturated fats in Mediterranean countries but also an important source of several micronutrients and food components. It appears to be a favourable indicator of the risk of various common cancers, particularly of the (upper) digestive tract but also of the colorectum, breast and ovary.

# Sub article: Nursing assessment as part of a multidisciplinary approach to nutritional assessment

**Published:** 2010

**Authors:** G Magon

**Affiliations:** Servizi Assistenziali, European Institute of Oncology, Milan, Italy

In this report, I will aim to define the characteristics of the nursing appraisal that facilitates a nutritional assessment of cancer patients.

I will begin with some important quotes, the first being one from Florence Nightingale (this year marking the one hundredth anniversary of her death) in 1860 in her publication ‘Notes on Nursing’, a founding text for modern nursing care (so much so that the introduction reads ‘What it is, and What it is not’), devotes an entire chapter to ‘Taking Food’.

Virginia Henderson in the declination of what constitutes the thinking behind the production of theory (of the most notable in International circles), identifies 14 nursing care needs that are common to every human being, which include eating and drinking.

We can, therefore, say that the concept of nutritional needs is historically recognised as part of specific nursing interventions, which are to be activated where required or where potentially required. Today, pre-registration nursing courses require the study of nutrition, and the documentation systems require detailed records of the characteristics of that need.

Time passes quickly and looking at the present day and the context here in Lombardy, we cannot fail to note, always more stringently, that health care organisations, and their employees are required to demonstrate (through compliance with standards) the processes to which we subject our patients (or to put it as Virginia Henderson—the beneficiaries of our intervention). The definition of specific standards for nutrition and nutritional therapy (e.g. AOP 1.6, COP 5, PFE 4, etc.) are an example.

I have deliberately quoted the context of Lombardy for the implementation of the standards of Joint Commission, but I must mention the urban context as the Local Health Agency of Milan has adopted the Guidelines in the Prevention of Malnutrition.

With all of these requirements, comes the task of analysing patient’s notes regarding aspects that make reference to diet, supplemented by research to develop a thesis of a student dietician with the same goal.

In agreement with the Dietetic service of the EIO, we decided to deal with this issue by allowing a multidisciplinary team, including nurses and dieticians, to move from the concept of identification of nutritional needs, towards an assessment of the nutritional status of the oncology patient. This requires a multidisciplinary approach in which there is clarity regarding completeness and accuracy of data to be collected, definition of responsibility and definition of timeframes (in this case, there is the necessity to consider continuity of care). With regards to completeness and accuracy of data collection, we have included a focus of interest on the evaluation of nutritional status in the process that has led to the development of new nursing documentation (today paper, tomorrow computerized and integrated with other health care professionals notes). This project of revision anticipates the passage towards a documentation oriented to the identification of a Nursing Minimum Data Set (NMDS) and to the identification of existing specific references to nutritional status (e.g. weight loss in the last three–six months, glucose intolerance, etc.). The working group, drawing on the scientific production by the Oncology Nursing Association has led to the development of an NMDS of 48 outcomes, grouped into 13 categories, one of which is that of NUTRITIONAL STATUS.

In relation to the accuracy of data collection, we have focused the discussion on what are the necessary data to investigate and in which phases of the nursing assessment. We have identified the need to investigate normal weight, weight changes in the last three months, calculation of the body mass index, capacity/limitations in the assumption of food, reporting of food intake in order to cover calorific needs and the definition of ‘items’ in order to activate a dietetic consultation/referral. For the definition of this last aspect, we made a choice that was in line with the policy and characteristics of the EIO and have used the terminology of the Common Terminology Criteria for Adverse Events (CTCAE version n°4) from the National Cancer Institute (USA).

The definition of levels of responsibility is a direct consequence of the adoption of valid nursing documentation (Palese *et al* 2006) a new procedure has also been developed (DIE.PG.0087.D) relating to diet and clinical nutrition, in which responsibilities are described (point 4) and the operating procedures are outlined (point 5), as well as the criteria for activating referral to the dietician (point 5.1). The introduction of the Primary Nursing model of care (the project started in October 2010) is a cornerstone in the concept of taking care of the person in need and in the planning of nursing care, both of which relate to individual responsibility not only with treatment (of particular interest to the medico legal representative) but, above all, in treatment outcomes.

Finally, we have considered the theme of time frames, or ‘What time is appropriate to enable the assessment of nutritional requirements’; in EIO, this is already active during pre-operative outpatient visit with the participation of the Nursing Case Manager in the detection of weight loss (or also weight gain in some cases), reporting restrictions on food intake and consequent activation of a dietician referral and counselling to allow corrective interventions.

During hospitalization, supervision is provided as outlined previously, while during discharge, multidisciplinary collaboration is fundamental to initiate an effective health education intervention relating to the clinical condition of the patient.

During the outpatient phase, patients continue to undergo assessment in the detection of outcomes relating to the dietetic consultation and multidisciplinary collaboration (important collaboration with the enterostomy). We have provided a telephone follow-up service for hospitalized patients, but, above all, we aim for more, in terms of completeness of the service, activation of a 24-h outpatient service—this service was created with the goal of allowing direct access to the EIO, in case of urgent problems, without having to pass via a general medical doctor. This provides access for patients previously treated at the Institute, which include also those patients with problems relating to nutrition and to the use of safeguards to promote food intake.

Palese A, Cabarcas GR, Dotti R and Riboli O. Documentazione scritta (consegne e piani di assistenza). Assistenza infermieristica e ricerca 2006; 25: 109–114.

# Sub article: HEN study: home enteral nutrition in malnourished patients after major surgery for gastrointestinal malignancy

**Published:** 2010

**Authors:** S Colatruglio, F Valoriani

**Affiliations:** 1 Struttura Semplice di Nutrizione Clinica, National Tumour Institute, Milan, Italy; 2 Dietetic and Clinical Nutrition, European Institute of Oncology, Milan, Italy

## Background and rational

The prevalence of malnutrition in patients with gastrointestinal malignancy has been reported to range from 26% to 57%; weight loss is often present since the beginning of the disease and, frequently, the nutritional status further deteriorates throughout the treatments. Malnutrition is known to be a risk factor in the overall treatment of patients with gastrointestinal cancer, and it has a negative impact on the final prognosis [1]. Weight loss is associated with increased post-operative morbidity and mortality [2], duration and costs of hospital stay, a reduction in tolerance to chemotherapy and worsens quality of life [3]. Body mass index, recent weight loss and change in food intake have been demonstrated to correlate with changes in cellular function and clinical outcome; these parameters are used to calculate the nutritional risk score in order to identify patients who are likely to benefit from nutritional intervention [4]. After gastrointestinal surgery, patient’s body weight usually declines in the two months following hospital discharge [5]. Post-operative and post-discharge administration of oral nutritional supplements has been investigated in different clinical settings, in six randomised trials, with conflicting findings [4,6–10].

Enteral nutrition (EN), a cost effective tool, is demonstrated to reduce post-operative infectious complications in malnourished patients undergoing major gastrointestinal surgery [11]. EN can be administrated for long periods through a jejunostomy, a fine needle catheter that can be safely positioned during surgery [12–13]. Regular monitoring of nutritional status and continuation of enteral nutrition in patients after hospital discharge might prevent further deterioration of nutritional status.

## Objective and selection criteria

The aim of this study is to evaluate the efficacy of home enteral nutrition (HEN) on the nutritional status, the quality of life and tolerance to chemotherapy in malnourished patients who undergo major gastrointestinal surgery for malignancy. The primary objective is the maintenance of nutritional status after hospital discharge, and secondary objectives are the improvement in quality of life and tolerance to chemotherapy.

Inclusion Criteria: both genders over 18 years of age, documented cancer of the gastrointestinal tract (oesophagus, stomach, pancreas, biliary tract), candidate for major elective surgery, pre-operative nutritional risk score ≥ 3 and written informed consent.

Exclusion Criteria: Age < 18 years, Karnofsky index < 60, renal failure (ongoing haemodialysis or plasma creatinine > 3 mg/dl), respiratory failure (arterial blood PaO_2_ < 70 mmHg), ASA score = 4 or 5, Child-Pugh class C, short bowel syndrome, pregnancy, emergency operation and foreign residence or residence in Italian region with no regulation designed for home artificial nutrition.

## Study plan

General design issues: Phase III, mono-centre, controlled, open-label, two-parallel groups, randomized, clinical trial and the type of comparison is observation. The study is promoted by the National Cancer Institute and the European Institute of Oncology, Milan, Italy.

All patients fulfilling the selection criteria will be included and randomized in the study; the planned enrolment is 140 patients (70 in each group) over a period of two years. All patients will continue observation for six months after discharge. In all patients included in the study, a fine needle catheter jejunostomy will be implanted at the end of the scheduled surgery; NE will start on post-operative day 1 and progressively increase, with the objective of supplying 25 kcal/kg b.w. and 0.2–0.25 g nitrogen/kg b.w. daily. Before discharge patients will be randomized to receive either home enteral nutrition (treatment group) or nutritional counselling (control group). In the HEN group, the planned nutrition will cover the basal energy requirement calculated with the Harris Benedict formula, it will be administrated preferentially through an integration of oral diet. Enteral Nutrition will include any standard polymeric formula providing 1–1.5 kcal/ml with carbohydrates 50%–60%, lipids 25%–35% and protein 12%–20%. HEN can be withdrawn in the treated group, after two months from discharge, whenever a weight gain ≥ 5% is reported, and oral alimentation is regular and adequate. In the control group, specific nutritional indications including total energy and protein requirements will be provided to patients and high-calorie oral supplements will be prescribed whenever necessary.

The HEN can be started in patients of the control group if a further weight loss ≥5% is reported. Anthropometric, biochemical and functional measures will be used for assessment and monitoring of the nutritional status, before discharge, and one, two, four and six months after discharge. Quality of life is assessed at hospital admission and two months after discharge, using the self-administrated FAACT questionnaire; the tolerance to chemotherapy is evaluated by the ratio CT planned/CT administered; numbers and degrees of side effects were scored in accordance to WHO classification for toxicity.

## References

[1] Dewys WD, Begg C, Lavin PT (1980). Prognostic effect of weight loss prior to chemotherapy in cancer patients. Eastern Cooperative Oncology Group. AM J MED.

[2] Mullen JL, Gertner MH, Buzby GP (1979). Implications of malnutrition in the surgical patient. Arch Surg.

[3] Andreyev HJ, Norman AR, Oates J (1998). Why do patients with weight loss have a worse outcome when undergoing chemotherapy for gastrointestinal malignancies?. Eur J Cancer.

[4] Kondrup J, Allison SP, Elia M (2003). ESPEN guidelines for nutrition screening 2002. Clin Nutr.

[5] Beattie AH, Prach AT, Baxter JP (2000). A randomised controlled trial evaluating the use of enteral nutritional supplements postoperatively in malnourished surgical patients. Gut.

[6] Delmi M, Rapin CH, Bengoa JM (1990). Dietary supplementation in elderly patients with fractured neck of the femur. Lancet.

[7] Keele AM, Bray MJ, Emery PW (1997). Two phase randomised controlled clinical trial of postoperative oral dietary supplements in surgical patients. Gut.

[8] MacFie J, Woodcock NP, Palmer MD (2000). Oral dietary supplements in pre- and postoperative surgical patients: a prospective and randomized clinical trial. Nutrition.

[9] Espauella J, Guyer H, Diaz-Escriu F (2000). Nutritional supplementation of elderly hip fracture patients. A randomized, double-blind, placebo-controlled trial. Age Ageing.

[10] Smedley F, Bowling T, James M (2004). Randomized clinical trial of the effects of preoperative and postoperative oral nutritional supplements on clinical course and cost of care. Br J Surg.

[11] Bozzetti F, Braga M, Gianotti (2001). Postoperative enteral versus parenteral nutrition in malnourished patients with gastrointestinal cancer: a randomised multicentre trial. Lancet.

[12] Biffi R, Lotti M, Canciarelli S (2000). Complications and long-term outcome of 80 oncology patients undergoing needle catheter jejunostomy placement for early postoperative enteral feeling. Clin Nutr.

[13] Schwarz RE (2002). Simple feeding jejunostomy technique for postoperative nutrition after major upper gastrointestinal resection. J Surg Oncol.

# Sub article: Physical activity and cancer: epidemiologic evidences

**Published:** 2010

**Authors:** D Palli, B Bendinelli, G Masala, D Occhini, M Castaldo

**Affiliations:** Nutritional and Molecular Epidemiology Unit, Cancer Research and Prevention Institute (ISPO), Florence, Italy

In the last Expert Report ‘Food, Nutrition, Physical Activity and the Prevention of Cancer: A Global Perspective’, 2007), jointly produced from World Cancer Research Fund (WCRF) and the American Institute for Cancer Research (AICR), the results of a systematic review of the literature concerning risk factor for cancer together with a series of recommendations for cancer prevention are reported. The document recommends ‘physical activity as part of everyday life’ in order to reduce cancer risk. In particular, there is convincing epidemiologic evidence of a protective effect of physical activity on colon cancer risk, probable evidence for breast (in post-menopausal women) and endometrium cancer risk and suggestive evidence for lung, pancreas and breast (pre-menopause) cancer risk. More recent literature confirms the opinions of the WCRF/IACR expert panel.

In addition to the general beneficial effect of weight loss and change in body composition mediated by physical exercise, other specific and independent mechanisms were hypothesized for the association between physical activity and reduced risk for several kinds of cancer. The influence of physical activity on sexual steroid hormones (breast, endometrium, prostate and colorectal cancer), on metabolic hormones (breast, colon and pancreatic cancer), on gut motility (colon and pancreatic cancer) and on immuno-modulation (breast and endometrium cancer) was discussed.

In epidemiological studies, three main classifications of physical activity are used:

- type of physical activity (occupational, household, transport and recreational);
- intensity of exercise indicated by metabolic equivalent, ratio between the energy spent by a subject during an activity and the resting metabolic rate in the same time;
- physical activity level, mean level of physical activity during a day (ratio between the daily total energy expenditure and the basal energy expenditure).

In these types of studies, physical activity is rarely measured with precision. Physical activity questionnaires (PAQs) are the tools most frequently reported in the literature; however, some concerns emerged about the use of PAQs:

- there are differences among studies as to what is considered ‘physical activity’ (recreational, sports, working ...);
- in the literature, different kinds of PAQ are used, thus the comparison between studies is quite difficult;
- physically active people tend to be more *health conscious* (non smokers, healthy diet) than sedentary people, thus the collection of these confounding factors is mandatory in studies on the association between physical activity and cancer risk;
- people tend to report vigorous and recreational activity more accurately than other kinds of activities (reporting bias).

Instruments for a more objective measurement of physical activity include: pedometers, heart rate monitors and accelerometers. A good estimation of the total daily physical activity may be, however, achieved by the simultaneous use of a heart rate monitor and an accelerometer (Actiheart monitor).

A physical activity and diet intervention trial to reduce mammographic breast density in post-menopausal women (a strong risk factor for breast cancer) is ongoing in the Nutritional and Molecular Epidemiology Unit. It is a factorial randomized trial conducted in post-menopausal women at increased risk of breast cancer (breast density > 50% in mammographic examinations performed in the frame of the local screening program). A structured 24-month intervention is performed in order to evaluate the effectiveness of reducing mammographic density, of an increasing in moderate exercise and/or of a modification in the diet according to the WCRF 2007 recommendations.

## Conclusions

Today, it is scientifically proven that physical inactivity is a key risk factor for chronic diseases including cancer, but a growing number of people are not achieving the levels of physical activity recommended for a good health. In cancer control research related to physical activity it seems necessary to focus on understanding the type and amount of physical activity that brings the greatest benefit in terms of primary prevention. We also need to determine how to increase physical activity in populations and how to improve the performance of ‘measurement methods’ to be used in large-scale studies.

# Sub article: Lung cancer and diet: preliminary results of the COSMOS study

**Published:** 2010

**Authors:** P Gnagnarella, G Veronesi, R Bertolotti, M Bellomi, L Spaggiari, D Palli, P Maisonneuve

**Affiliations:** 1 Divisione Epidemiology and Biostatistics; 2 Department of Thoracic Surgery; 3 Department of Radiology, European Institute of Oncology, Milan, Italy; 4 Nutrition and Molecular Epidemiology Unit, Cancer Research and Prevention Institute (ISPO), Florence, Italy

## Introduction

Cancer of the lung is the most common type of cancer in the world [3]. The major risk factors are tobacco smoking followed by occupational exposures. Epidemiological research has provided evidence that dietary habits may play an important role in lung cancer aetiology [12]. The 2007 WCRF/AICR expert report concludes that increased fruit intake and food containing carotenoids probably protect against lung cancer, but there are only limited evidence suggesting that vegetables and foods containing selenium and quercetin protect against lung cancer. The association still remains unclear [2, 10] in smokers. There is also limited evidence suggesting that red meat, processed meat, total fat and butter are causes of lung cancer [12].

As part of the COSMOS study (Continuous Observation of Smoking Subjects) designed to implement a comprehensive screening programme for early diagnosis of lung cancer in high-risk volunteers in North West Italy [11], we assessed dietary intake of participants using a semi-quantitative food frequency questionnaire. In this study, we report the preliminary results examining the association between nutrients intake and lung cancer risk in high-risk volunteers.

## Materials and methods

The COSMOS study is a single-centre non-randomized lung cancer screening trial, conducted in Northern Italy. Details of participants, screening protocol and diagnostic work up have been published elsewhere [11]. Briefly, we enrolled asymptomatic volunteers, age 50 years or more, who were current or former heavy (≥20 pack-years) smokers in 2004. Ex-smokers had stopped not more than ten years previously. Those with malignant disease (except treated non-melanoma skin cancer) or a diagnosis of malignant disease within the previous five years were excluded. All recruited volunteers gave written consent to receive annual low-dose spiral computed tomography (LD-CT) for five consecutive years. They also compiled a self-reporting questionnaire investigating respiratory symptoms, smoking habits, exposure to other risk factors, medical and family history. The study was approved by the ethics committee of the European Institute of Oncology.

## Dietary assessment

At baseline, usual diet before the enrolment was measured by a semi-quantitative food frequency questionnaire originally developed for the EPIC-Italy study [5, 6]. It is a validated self-administered instrument [7], estimating the daily intake of foods and nutrients, referred to the previous year. It contains 14 sections with 248 questions concerning 188 different food items, and 17 sets of pictures showing a small, medium and large portion size are also available. The absolute frequency of consumption of each item is assessed, reporting the number of times a given food item is consumed (per day, week, month or year). The consumption of vitamins and minerals supplements has also been collected to complete the nutrient intake of volunteers. Participants reported the brand name, the frequency of consumptions per day or per week and the numbers of months, referred to the previous year.

## Statistical analysis

The average daily quantities of foods, energy and nutrients consumed by participants have been calculated using a computer program (Pale et al, 2003), non-including supplement intake. Nutrient intake has been calculated according to a specifically developed Italian food composition database for epidemiological study [8].

Cox proportional Hazards regression was used to analyse the association between nutrients intake and risk of lung cancer. We divided the consumption of nutrients (macro- and micronutrients) according to quartiles of consumptions. Multivariate models were adjusted for age, sex, smoking status, number of pack-years, past history of emphysema or chronic obstructive pulmonary disease (COPD) and total energy intake.

## Results and conclusion

During 19,616 person-years of observation, 134 participants of the COSMOS study were diagnosed with lung cancer. Initially, 5203 subjects were enrolled, but dietary information was obtained for 4363 participants (84%). After exclusion of patients with abnormal dietary values (outliers), the final study population included 4337 subjects (Table 1). Median age at baseline was 57 years (50–84), 20% of subjects were ex-smokers and 80% were current smokers. The percentage of supplement users was (15%, 648 subjects) higher compared with the Italian food consumption survey (5%) or with other European studies (3%–8%) [1].

Mean energy intake reported by the study population was 2254 kcal (9428 kJ) per day (Table 2). Protein contributed on average for 16% of food energy, fat for 34%, carbohydrates for 43% and alcohol for 8%. We observed a higher intake of total fat, total saturated fatty acids (FSA) (12%), cholesterol (353 mg), soluble carbohydrate (17%) and a low intake of dietary fibre (19 g), compared to the national recommendations [4]. These data are in line with the results of the last Italian National Food Consumption Survey (INRAN-SACI 2005) [9]. On average, the intake for mineral and vitamins were adequate to cover the recommendations, except for women who did not reach recommended levels of calcium and vitamin D. The data indicate that our high-risk population had a diet poorer in anti-oxidant vitamins (mainly in β-Carotene and vitamin E) compared to the National Survey [9].

In our study, no significant association was found between either macro- or micronutrients and lung cancer risk, when we compared the highest versus lowest quartiles of consumption (Table 3). The evidence of the association between nutrients and lung cancer risk are actually scarce in literature. No conclusion can be drawn for macronutrients, dietary fibre; alcohol, vitamins (vitamin A; thiamine; riboflavin; niacin; vitamin B6; folate; vitamin C; vitamin E), minerals (calcium; copper; iron; zinc) and other bioactive compounds (pro-vitamin A carotenoids, lycopene, flavonoids) as reported by the 2007 WCRF/AICR expert report. Only carotenoids (from foods) probably protect against lung cancer. Limited is the evidence for foods containing selenium and total fat, but our results do not support these from previous studies and do not help to understand the association with other components included in our analysis.

The null association reported may be explained in part by the strong association between lung cancer and smoking, influencing the effect of the single nutrients. In addition, smokers tend to have a less healthy diet and to be leaner than non-smokers, but our population results to be health conscious. We found a high percentage of supplement users, and overweight and obese (together 56%). These factors may have influenced our results. Further analysis needs to investigate the associations between dietary nutrients and the risk of lung cancer risk in subgroups (sex, smoking status, histological subtype o) in our population. To complete the analysis, we will also include nutrients intake (vitamins and minerals) coming from supplements.

## References

[1] Cho E, Hunter DJ, Spiegelman D, Albanes D, Beeson WL, van den Brandt PA, Colditz GA, Feskanich D, Folsom AR, Fraser GE, Freudenheim JL, Giovannucci E, Goldbohm RA, Graham S, Miller AB, Rohan TE, Sellers TA, Virtamo J, Willett WC, Smith-Warner SA (2006). Intakes of vitamins A, C and E and folate and multivitamins and lung cancer: a pooled analysis of 8 prospective studies. Int J Cancer.

[2] Feskanich D, Ziegler RG, Michaud DS, Giovannucci EL, Speizer FE, Willett WC, Colditz GA (2000). Prospective study of fruit and vegetable consumption and risk of lung cancer among men and women. J Natl Cancer Inst.

[3] Garcia M, Jemal A, Ward EM, Center MM, Hao Y, Siegel RL, Thun MJ (2007). Global Cancer Facts & Figures 2007.

[4] LARN (1996).

[5] Pala V, Sieri S, Palli D, Salvini S, Berrino F, Bellegotti M, Frasca G, Tumino R, Sacerdote C, Fiorini L, Celentano E, Galasso R, Krogh V (2003). Diet in the Italian EPIC cohorts: presentation of data and methodological issues. Tumori.

[6] Palli D, Berrino F, Vineis P, Tumino R, Panico S, Masala G, Saieva C, Salvini S, Ceroti M, Pala V, Sieri S, Frasca G, Giurdanella MC, Sacerdote C, Fiorini L, Celentano E, Galasso R, Decarli A, Krogh V (2003). EPIC-Italy. A molecular epidemiology project on diet and cancer: the EPIC-Italy Prospective Study. Design and baseline characteristics of participants. Tumori.

[7] Pisani P, Faggiano F, Krogh V, Palli D, Vineis P, Berrino F (1997). Relative validity and reproducibility of a food frequency dietary questionnaire for use in the Italian EPIC centres. Int J Epidemiol.

[8] Salvini S, Parpinel M, Gnagnarella P, Maisonneuve P, Turrini A (1998). Banca Dati di Composizione degli Alimenti per Studi Epidemiologici in Italia.

[9] Sette S, Le Donne C, Piccinelli R, Arcella D, Turrini A, Leclercq C (2010).

[10] Touvier M, Kesse E, Clavel-Chapelon F, Boutron-Ruault MC (2005). Dual Association of beta-carotene with risk of tobacco-related cancers in a cohort of French women. J Natl Cancer Inst.

[11] Veronesi G, Bellomi M, Mulshine JL, Pelosi G, Scanagatta P, Paganelli G, Maisonneuve P, Preda L, Leo F, Bertolotti R, Solli P, Spaggiari L (2008). Lung cancer screening with low-dose computed tomography: a non-invasive diagnostic protocol for baseline lung nodules. Lung Cancer.

[12] World Cancer Research Fund / American Institute for Cancer Research (2007). Food, Nutrition, Physical Activity, and the Prevention of Cancer: a Global Perspective.

# Sub article: Nutrition and Tumours

**Published:** 2010

**Authors:** F Berrino

**Affiliations:** Department of Predictive and Preventive Medicine, National Tumour Institute, Milan, Italy

## Background

Breast cancer (BC) is the most common malignancy in women and is their leading cause of death from cancer. In high-income countries, the incidence of BC has increased steadily over the past decades, but BC mortality is declining, suggesting a benefit from early detection and more effective treatment. BC survivors are constantly increasing, and research investment for the identification of modifiable factors associated with BC recurrences is increasing too. Western lifestyle, characterized by low levels of physical activity and a diet rich in refined carbohydrates, animal fats and protein is associated with high prevalence of metabolic syndrome, insulin resistance and high serum levels of sex hormones and growth factors. All these factors are strongly related with BC risk and BC prognosis but are potentially modified through diet and lifestyle.

## Method

The present work summarizes the metabolic, hormonal and dietary correlates of increased risk of BC and BC recurrences.

## Results

Obesity is associated with an increased risk of BC after menopause, while no association or slightly reduced BC risk has been found before menopause. Preventing weight gain in adulthood, however, would decrease the overall burden of BC. Obesity has been shown to adversely affect prognosis in both pre- and post-menopausal BC, after controlling for clinical and pathological prognostic factors. High serum levels of steroid sex hormones and of bio-available insulin-like growth factor I (IGF-I) are associated with an increased risk of BC. Furthermore, insulin and markers of insulin resistance, such as abdominal obesity, high blood glucose, high serum level of testosterone, and metabolic syndrome, may affect both BC incidence and prognosis. Sedentary lifestyle is associated with increased BC risk, both before and after menopause. Women who practice regularly at least some physical activity decrease their BC risk by 30% or more, and there is increasing evidence that physical activity may protect also against BC recurrences. The association between dietary fat intake and BC is an highly controversial topic in epidemiology. Results of the Women Initiative on Nutrition Study (WINS), a randomized dietary prevention trial, suggested a strong benefit of dietary fat reduction on relapse-free survival with the strongest protection for hormone receptor-negative BC. The Women’s Healthy Eating and Living (WHEL) Study tested whether a dietary pattern high in vegetables, fruit and fiber and low in fat might reduce BC relapses in women with early-stage BC. The primary analyses of the WHEL study did not demonstrate an event-free survival advantage in patients randomized in the dietary arm. This trial, however, was isocaloric and the intervention group did not loose weight.

## Conclusion

The briefly reviewed factors are strongly related to diet and lifestyle and may be potentially modified. Our DIANA (Diet and Androgens) intervention trials demonstrated that a sustainable dietary modification aimed at lowering insulin levels, based on Mediterranean and macrobiotic dietary principles, can reduce body weight, metabolic syndrome and the bioavailability of sex-hormones and growth factors. Together with other studies showing that Mediterranean diet can revert metabolic syndrome, these results suggest that dietary changes should be recommended for both BC prevention and treatment.

# Sub article: Dietary patterns and cancer in Italy and Europe

**Published:** 2010

**Authors:** V Krogh, C Agnoli

**Affiliations:** National Tumour Institute, Milan, Italy

Traditional analyses in nutritional epidemiology typically examine the relationship between diet and diseases focusing on single nutrients or foods. However, due to limitations in this approach, some decades ago a new and more holistic way to look at the association between diet and health spread: the dietary pattern approach. The main reasons for this shift are:

- persons do not eat isolated nutrients or single foods, but a wide variety of foods with combinations of nutrients that may be synergistic;
- high correlation between nutrients make it difficult to study their separate effects;
- the effect of a single nutrient may be to small to be detect;
- analyses based on a large number of nutrients or food items may produce statistically significant associations simply by chance,
- since nutrients intake is associated with some dietary patterns, results based on this analysis could be confused by the effect of the dietary pattern.

Dietary patterns, which take into account cumulative and interactive effects among dietary components and reflect real-world dietary preferences, may be particularly suitable when several dietary components are involved in the onset of a disease.

In dietary pattern analysis, the collinearity of nutrients and foods could be exploited with advantage because dietary patterns are characterized on the basis of eating behaviour; furthermore, the study of dietary patterns could have important implications because they are easy to translate into dietary guidelines.

## Methods for defining dietary patterns

**[17]**

There are two main approaches to define dietary patterns (see Figure 1):

- ‘*a priori*’ dietary patterns or diet quality scores: they consist of nutritional variables (foods and/or nutrients), thought to be important for the health or in a specific cultural context, that are quantified and summed to provide a comprehensive measure of dietary quality;
- ‘*a posteriori*’ dietary patterns: they are obtained trough statistical modelling of the data at hand.

## *A priori*-defined dietary patterns

This kind of dietary patterns reflect the current knowledge on the relationship between diet and a specific disease and can be easy translated into country-specific or culture-specific nutritional recommendations. They can be based on dietary guidelines, on available scientific evidence on the relationship between diet and a specific disease, or on a specific dietary tradition.

Several key issues should be keep in mind when constructing a diet quality score:

- choice of the components to include in the score;
- choice of the nutritional variables to include in each component;
- choice of the cut-off values;
- exact quantification of the pattern components;
- whether adjusting or not for energy intake;
- decision on the relative contribution of the individual components to the total score.

Several diet quality scores have been developed worldwide; below there is a brief explanation of the main scores developed in Italy and in Europe and the findings on the relationship between adherence to them and cancer risk.

### *Mediterranean diet score* [21]

Scoring is based on intake of 9 items (see Table 1): vegetables, legumes, fruit and nuts, dairy products, cereals, meat and meat products, fish, alcohol and the ratio of monounsaturated to saturated fat. For most items, consumption above study median receives 1 point; all other intakes receive 0 points. For dairy products, meat and meat products, consumption below the median receives 1 point. Medians are gender specific. For ethanol, men who consumed 10–50 g/day and women who consumed 5–25 g/day received 1 point; otherwise, the score was 0. The range of possible scores is 0 to 9.

Previous versions of this score included potatoes in the vegetables component and did not include fish [20].

### *Mediterranean adequacy index* [8]:

This index is calculated as the ratio between consumption of typical Mediterranean food items and consumption of less typical Mediterranean food items.

Mediterranean foods include: bread, cereals, legumes, potatoes, vegetables, fruit, nuts, fish, wine and vegetable oils.

Non-Mediterranean foods include: milk, cheese, meat, eggs, animal’s fats and margarines, sweetened beverages, sweets and sugar.

Food items can be expressed as percentage of energy intake, g/day, g/1000 kcal, or g/4.2 MJ.

### *Healthy diet indicator* [9] (see Table 1):

Developed on the basis of the WHO dietary recommendations for the prevention of several chronic diseases, it included nine components (see Table 2): saturated fats, polyunsaturated fats, protein, complex carbohydrates, fiber, fruit and vegetables, pulses/nuts/seeds, monosaccharides and disaccharides, cholesterol. For each component, the score is 1 if the intake meets to the recommendations, otherwise it is 0. The range of possible score is 0 to 9.

### *Food-based quality index* [10]:

This index is based on the WHO dietary guidelines and includes as components seven food items: fruit and nuts, vegetables, cereals, meat, milk, potatoes and cheese. For each component, the score is 1 if the intake meets to the recommendations, otherwise it is 0. The range of possible score is 0 to 7.

### *Healthy food index* [14]:

It is calculated by assigning one point to each of the following characteristics:

- not consuming butter, lard or margarine daily;
- consuming raw or boiled vegetables at least once a day;
- consuming whole bread at least once a day;
- consuming fruit at least once a day.

Possible scores, therefore, range from 0 to 4.

### *Food pyramid index* [12]:

This index is calculated as the ratio between more fatty food items of the Group 1 of the Italian Food Pyramid (i.e. oils, fats, sweets, dairy products, red meat, poultry and fish) to those with negligible fat content of Group 2 (i.e. pasta, rice, bread and legumes). Vegetables and fruit were not included.

## Relation between *a priori* dietary patterns and cancer risk

The Mediterranean diet has gained considerable attention recently, and adherence to this diet has been studied extensively. Therefore, the vast majority of study investigating the relationship between an *a priori* dietary pattern and cancer risk in Europe applied the Mediterranean diet score. Adherence to this index was found to be inversely associated to:

- post-menopausal breast cancer incidence [22];
- gastric adenocarcinoma incidence [4];
- upper aero-digestive tract cancer incidence [3];
- all cancer incidence [1];
- all cancer mortality [21].

## Strengths and limitations of *a priori* dietary patterns

Strengths of *a priori* dietary patterns include the following characteristics:

- analytically simple to compute;
- easily reproducible and comparable;
- usually associated with health outcomes;
- they may be formulated to investigate the effects of diet on specific diseases.

On the other hand, they have some limitations:

- those that dichotomize components (e.g. above vs below the median intake) do not take into account the full range of amounts of foods consumed (problem of the extremes of the distribution);
- subjectivity is introduced in the choice and quantification of components;
- they may not adequately deal with the correlations in intake of various dietary factors and nutrient–nutrient interactions;
- limited by available scientific knowledge.

## *A posteriori*-defined dietary patterns

In data-driven approaches, a large set of dietary variables are aggregated and reduced to a smaller set of variables. These methods do not require *a priori* theory on which components should be included in the dietary pattern and on their quantification. Two main approaches can be applied to derive *a posteriori* dietary patterns:

- factor analysis, based on intercorrelations between dietary variables;
- cluster analysis, based on individual differences in mean intakes of dietary constituents.

### *Factor analysis*:

Factor analysis is a multi-variate statistical technique, which identifies food items that are usually consumed together: it aggregates specific dietary variables in factors on the basis of the degree to which the variables are correlated to one another in the dataset. The resulting factors represent distinct dietary patterns, they are uncorrelated with each other and are linear combinations of the observed dietary variables that explain the variance in the data.

Factors are not mutually exclusive: on the basis of their diet, individuals receive factor scores for each derived factor or pattern. Factors are continuous variables that are often categorized into quantiles.

### *Cluster analysis*:

Cluster analysis is a multi-variate statistical technique that aggregates individuals into relatively homogeneous subgroups (clusters) with similar diets.

Dietary patterns are derived on the basis of differences in mean intakes between subjects, individuals belong to one cluster only. Clusters are categorical (nominal) variables.

## Reduced rank regression

Analytical technique that aims at identifying linear functions of predictors (e.g. foods) that explain as much variation as possible in a set of intermediate response variables (e.g. nutrients or biomarkers related to the outcome of interest). The resulting factors can then be examined in relation to diseases.

It is a mix between *a priori*-defined and *a posteriori*-defined dietary patterns because it uses both dietary data at hand and prior knowledge for choosing appropriate response variables.

## Reproducibility and validity of *a posteriori* dietary patterns

Although *a posteriori* dietary patterns could not be exactly the same across studies since they depend on available data, major patterns show reasonable reproducibility. Furthermore, validation studies found reasonable correlations between data-driven patterns and nutrients or biomarkers.

Several *a posteriori*-defined dietary patterns have been identified in European and Italian studies.

## Italian studies

### *ORDET cohort (factor analysis)* [18,19]:

- salad vegetables: raw vegetables and olive oil;
- western: potatoes, red meat, eggs and butter;
- canteen: pasta and tomato sauce;
- prudent: cooked vegetables, legumes, fish and low-alcohol consumption.

### *EPIC-Italy Elderly cohort (factor analysis)* [13]:

- prudent: cooked vegetables, legumes and seeds oil;
- pasta and meat: pasta and other grains, tomato sauce, red and processed meats, animal fats, bread and wine;
- olive oil and salad: olive oil, raw vegetables, soups and poultry;
- sweet and dairy: added sugars, sweets, coffee, eggs, butter and milk.

### *Multicentric case-control study (factor analysis based on nutrients)* [6,2,7]:

- animal products: animal protein, animal fats, calcium, cholesterol, saturated fats, riboflavin, zinc and phosphorus;
- vitamins and fiber: vitamin C, fiber, folate, potassium, β-carotene equivalents, soluble carbohydrates and vitamin B6;
- unsaturated fats: vegetable fats, vitamin E, monounsaturated and polyunsaturated fats;
- starch-rich: starch, vegetable protein and sodium.

## European studies

*DIETSCAN pooling cohorts study (factor analysis)* [5,11]: It is remarkable that this pooling of four cohort studies found the same two *a posteriori* dietary patterns across the European studies included:

- vegetables: vegetables, legumes, citrus fruits, berries, pasta, rice, poultry, fish and oils;
- pork, processed meat and potatoes: pork meat, processed meat, potatoes and coffee.

### *EPIC-Potsdam cohort (reduced rank regression)* [16]:

- high fat consumption pattern (breast cancer): low consumption of bread and fruit juices, high consumption of processed meat, fish, butter and other animal fats and margarine.

## Relation between *a posteriori* dietary patterns and cancer risk

Table 3 below shows the main findings on the relationship between *a posteriori* dietary patterns and cancer risk:

In summary, ‘salad vegetables’ pattern was associated with reduced breast cancer risk, especially HER2 negative breast cancer [18,19]. ‘Animal products’ was associated with increased risk of breast [6] and gastric cancer [2]. ‘Unsaturated fats’ increased breast cancer risk [6] but reduced colon cancer risk [7], while ‘starch-rich’ increased the risk of breast, ovarian [6], gastric [2], colon and rectum cancer [7]. ‘Vitamins and fiber’, instead, was found to be protective for ovarian [6], gastric [2] and rectum cancer [7]. Another pattern based on nutrient, labelled ‘vitamin-rich’ and very close in composition to ‘vitamins and fibre’, was found to be protective for gastric cancer, while the same study found that a ‘traditional’ pattern, characterized by high intake of total protein, starch, alcohol, nitrite and N-nitrosodimethylamine, increased the risk of gastric cancer [15]. Finally, ‘high-fat consumption pattern’ was associated with increased breast cancer risk.

## Strengths and limitations of *a posteriori* dietary patterns

Strenghts of *a priori* dietary patterns include the following characteristics:

- they allow for biologic interactions among nutrients;
- they describe how people really eat;
- less influenced by *a priori* knowledge on the relationship between diet and diseases.

On the other hand, they have the following limitations:

- they do not necessarily represent ’healthy’ versus ‘unhealthy’ patterns;
- they may not be appropriate to predict a specific disease;
- limited data on the reproducibility and the validity of the methods;
- subjectivity is introduced in the grouping of dietary items, treatment of dietary variables, how to deal the gender differences, how to deal with energy adjustment, selecting a final pattern solution.

## Major challenges in dietary patterns analysis

A major challenge in studying the relationship between dietary patterns and risk of a specific disease is the selection of the best method to quantify dietary patterns. Furthermore, because they are composed with a variety of foods and/or nutrients, they are not informative about biological mechanisms underlying the association between dietary habits and disease.

Dietary patterns are likely to vary according to gender, culture, ethnic group and socio-economic status; therefore, results should be replicated in diverse populations. In addition, because of changes in food preferences and availability, patterns could also change over time.

## Conclusion

To conclude, dietary patterns analysis is a recent method proposed to study the relation between a complex exposure, i.e. diet and disease risk. This approach has many advantages, but because of its limitations, it will not replace analysis based on foods and nutrients; instead, it could be useful to complement more traditional analysis. In addition, a dietary pattern could also be assessed as a covariate when examining the effect of a specific nutrient on disease risk.

## Figures and Tables

**Figure 1.** Methods for defining dietary patterns (modified from Schulze & Hoffmann, *Br J Nutr* 2006)

**Table 1.** Components of the Mediterranean diet score and scoring system

**Table 2.** Components of the Healthy Diet Indicator and scoring system. Values are percentage of energy intake unless indicated (modified from Huijbregts, *BMJ* 1997)

**Table 3.** Association between a posteriori dietary patterns and cancer risk

## References

[1] Benetou (2008). Br J Cancer.

[2] Bertuccio (2009). Cancer Epidemiol Biomarkers Prev.

[3] Bosetti (2003). Cancer Epidemiol Biomarkers Prev.

[4] Buckland (2010). Am J Clin Nutr.

[5] Dixon (2004). Am J Clin Nutr.

[6] Edefonti (2008). Int J Cancer.

[7] Ferraroni (2010). Cancer Causes Control.

[8] Fidanza (2004). Nutr Metab Cardiovasc Dis.

[9] Huijbregts (1997). BMJ.

[10] Lowik (1999). Br J Nutr.

[11] Mannisto (2005). Cancer Causes Control.

[12] Massari (2004). Prev Med.

[13] Masala (2007). Br J Nutr.

[14] Osler (2001). Br J Nutr.

[15] Palli (2001). Cancer Causes Control.

[16] Schulz (2008). Br J Nutr.

[17] Schulze, Hoffmann (2006). Br J Nutr.

[18] Sieri (2004). Cancer Epidemiol Biomarkers Prev.

[19] Sant (2007). Int J Cancer.

[20] Trichopoulou (1995). BMJ.

[21] Trichopoulou (2003). N Eng J Med.

[22] Trichopoulou (2010). Am J Clin Nutr.
